# Effects of a Ketogenic Diet on Symptoms, Biomarkers, Depression, and Anxiety in Parkinson’s Disease: A Case Study

**DOI:** 10.7759/cureus.23684

**Published:** 2022-03-31

**Authors:** Melanie Tidman

**Affiliations:** 1 Preventive Medicine/Metabolic Health, A.T. Still University of Health Sciences, Albuquerque, USA

**Keywords:** neurodegenerative disease, metabolic health, integrative and functional nutrition, low carb, ketogenic diet, symptoms, anxiety, depression, biomarkers, parkinson's disease

## Abstract

The ketogenic diet has grown in popularity as an alternative or adjunct to medication therapy for Parkinson's disease (PD) and other neurodegenerative diseases (NDD). Traditional medication therapies often fail to produce desired improvements in PD symptoms and can have little or no effect on symptoms of depression and anxiety that often accompany a PD diagnosis. We document a case study involving a 68-year-old female with PD stage I and a history of mild symptoms of anxiety and depression. The subject adopted a traditional ketogenic diet (fats 70%; protein 25%; carbohydrates 5%) for 24 weeks. Baseline, 12-week and 24-week biomarkers (lab results), and scores on a depression scale, anxiety scale, and the Unified Parkinson's Disease Rating Scale (UPDRS) (parts I-III) for PD symptoms were compared. Significant improvements were observed in all health biomarkers, including a reduction in HbA1C, C-reactive protein (CRP), triglycerides, and fasting insulin, along with weight loss and reduction in cardiac risk factors. Improved high-density lipoprotein (HDL) levels were seen at 12 weeks and 24 weeks, along with improved anxiety symptoms at the 12-week and 24-week mark. Minimum improvement was seen on depression scale scores at 24 weeks. Based on our findings, the ketogenic diet is safe and effective for improving biomarkers of health, symptoms of depression, anxiety, and PD symptoms in patients with stage I PD. We recommend further clinical trial studies for more generalizable results.

## Introduction

Several research studies on new and alternative therapies as adjuncts to levodopa medications to manage both motor and nonmotor symptoms of Parkinson's disease (PD) have emerged. There is a growing interest in the investigation of nutritional approaches to managing PD. According to some studies, a low-fat, high-carbohydrate diet positively affects the dopamine precursor tyrosine, by triggering an insulin-induced rise in brain dopamine [1]. Additionally, the role of blood ketones and positive influences on brain energy metabolism have been explored in terms of specific applications for the improvement of motor and nonmotor symptoms in PD [1].

PD is the most commonly occurring neurodegenerative disease (NDD), with clinical presentations that can be heterogeneous. PD is characterized by specific motor signs and symptoms, loss of neurons in the substantia nigra pars compacta (SNpc), and the presence of Lewy bodies and neurites [2]. Generally, the motor symptoms include motor impairment, tremors, and balance and gait abnormalities; various nonmotor symptoms such as speech and sleep disturbances are also observed. Levodopa (l-Dopa) is the most widely used medication in treating PD but it can lead to various side effects, including the development of motor fluctuations and dyskinesias and nonmotor disturbances such as poor sleep quality [3]. According to Bloem et al. (2015), examples of motor symptoms that can respond poorly to medication management include speech impairments, balance and postural instabilities, and gait disturbances, including freezing [4]. Increased disability can arise from nonmotor symptoms, including cognitive deficits, anxiety, depression, and interference with the activities of daily living. It can be controlled suboptimally with standard medication management [4,5]. Additionally, older adult brains require significant energy for cognitive functions compared with other organ systems. In PD, cognitive impairment can be a frequent symptom and it can also be associated with more rapid decline [6]. Alternative treatments, which are mostly non-medication-based, may yield more significant improvements in nonmotor symptoms such as cognitive impairments and related symptoms of depression and anxiety in people with PD. Research into the subject is currently demonstrating a greater interest in exploring dietary approaches to nonmotor symptom management [1].

Research regarding nutritional approaches to disease management has been on the rise as conditions become less responsive to medication management in some cases over time. One of the most popular dietary approaches in treating NDDs like PD is the low-carb/healthy-fat ketogenic diet (LCHF/KD). Some small, non-randomized clinical trials have indicated that therapeutic carbohydrate restriction may positively influence both motor and nonmotor symptoms in PD by easing the passage of the dopamine precursor (tyrosine) into the cerebrospinal fluid to trigger changes in dopamine production in the brain [1]. Modifying nutritional intake and supplementation may improve symptoms associated with PD, including insomnia, depression, constipation, dystonia, or dyskinesia, and prevent or stabilize cognitive decline [7]. However, much controversy still exists over the role of dietary fat and carbohydrates in the treatment of neurological conditions like PD. The LCHF/KD approach may improve cognition by triggering a metabolic shift from glucose metabolism to the primary use of ketones [8] to sustain energy demands for the brain and reduce systemic and neural inflammation, especially in older persons with PD [5]. Adults with NDDs who primarily consume a low-fat diet have lower high-density lipoprotein (HDL), low-density lipoprotein (LDL), and total cholesterol levels. However, it is widely known that HDL has a protective neurological effect, and some studies have suggested a possible protective effect on inflammation as in NDDs like PD [1]. This report discusses the case of a 68-year-old woman with PD stage I and symptoms of depression and anxiety. The research question under consideration is as follows: how does consuming an LCHF diet affect symptoms, biomarkers, depression, and anxiety in persons with PD over a period of 24 weeks?

## Case presentation

Description of participant baseline levels

This case study involves a 68-year-old female with PD stage I (Hoehn and Yahr) [9], mild PD motor symptoms, and a history of mild-to-moderate symptoms of anxiety and depression. Her current medications included carbidopa-levodopa ER 50-200 1x/day and occasional gabapentin 100 mgs for pain. The participant exercised with a personal trainer two to three times per week and is a Ph.D. student at a local university. She is married with grown children and is independent in terms of mobility without assistance. The initial interview was conducted via Zoom (Zoom Video Communications, Inc., San Jose, CA). The participant reported occasional sleep disturbances, mild motor symptoms during daily activities, mild-to-moderate feelings of depression and anxiety, and issues with blood glucose control. Baseline scores were obtained using the Center for Epidemiologic Studies Depression Scale-Revised (CESDR-R-20), the Parkinson's Anxiety Scale (PAS), and the Unified Parkinson's Disease Rating Scale (UPDRS), and for biomarkers including HbA1C, triglycerides, HDL, fasting insulin, C-reactive protein (CRP), weight, waist circumference, and several common markers of metabolic health.

The participant's height at baseline was 5'3", and her weight was 202 lbs with a waist circumference measurement of 46" (BMI=35.8). The score on the PAS at baseline was 23, reflecting mild-to-moderate symptoms of anxiety, as compared with a possible total score of 48 [10]. Per the CESDR-R-20 scoring criteria, persons with scores of less than 16 do not present with depressive disorders [10]. The score on the CESDR-R-20 was 42, indicating moderate symptoms of depression. The UPDRS scores were as follows: part I: 2; part II: 11; and the total score for parts I, II, and III was 31. The patient reported that 1-25% of her day was characterized by "off" periods, occasional nausea, and sleep disturbance. She also reported that she consumed a standard recommended diet based on the current US dietary guidelines with reduced fat but had a tendency to consume mostly processed foods and sugar. She admitted to historically having some issues with control of blood glucose and mild hypertension.

Dietary intervention

The patient was educated at baseline on the adoption of an LCHF/KD nutritional approach that included 70-75% fats, 20-25% protein, and 5-10% carbohydrates, similar to the macronutrient concept in a study by Phillips et al. (2019). Protein requirements were set between 0.8 and 1.0 g/kg of body weight for the potential enhancement of I-dopa absorption and to avoid high protein intake, which could exacerbate symptoms of dyskinesias in some people with PD [11]. Educational materials were provided, including a recommended food list, a 100-page cookbook with recipes and meal plans [1], and a KetoMojo blood glucose/ketone meter (Keto-Mojo, Napa, CA) for testing fasting blood glucose and blood ketone levels at home. Zoom live meetings were held at the participant's request, and email communication was used weekly as required to answer questions or provide clarification on the food lists or meal plans. The participant was encouraged to test her fasting blood glucose and ketones daily to establish dietary consistency with a view to achieving fasting blood glucose below 100 mmol and blood ketones between 0.5-3.0 mmol [12]. Blood glucose and ketone logs were provided to the study researcher to document dietary compliance.

Baseline biomarkers

A comparison of health biomarkers (HDL, fasting insulin, triglycerides, CRP, weight/BMI, waist measurement) between baseline readings and those from 12-week or 24-week lab results was conducted to note any benefits of the new dietary regimen. Table 1 compares biomarker results at baseline, 12 weeks, and 24 weeks. Initially, it was notable that the participant's HbA1C fell between the levels identified by the American Diabetes Association as the diabetic range (6.7%) [13]. Other notable baseline readings that indicated higher-than-desirable levels were fasting insulin (18.6 mIU/L), triglycerides (127 mg/dL), cardiac risk ratio (2.88), and weight (202 lbs). CRP is a marker to check for inflammation, determine the risk for heart disease, or evaluate the risk of a second heart attack [14]. The baseline level of CRP placed the participant at lower risk for a cardiac event (<2.0 mg/L) at 1.29 mg/l. At baseline, the participant had a BMI of 35.8 kg/m^2^ and a waist circumference of 46". According to Johns Hopkins guidelines, the diagnosis of metabolic syndrome is made if a patient has three out of five key risk factors, which include high triglycerides (>150 mg/dl), low HDL (<50 mg/dl), large waist circumference (>35" for women), elevated blood glucose (>100 mg/dl), and hypertension (>130/85 mmHg) [15]. Elevated levels of HbA1C, a waist circumference of more than 35", low HDL (44 mg/dl) [16], and reported hypertension (BP >140/90 mmHg) qualified the participant for a baseline diagnosis of metabolic syndrome.

**Table 1:** Comparison of all variables at baseline, 12 weeks, and 24 weeks CESDR: Center for Epidemiologic Studies Depression Scale; PAS: Parkinson's Anxiety Scale; UPDRS: Unified Parkinson's Disease Rating Scale; HDL: high-density lipoprotein; Hs-CRP: high-sensitivity C-reactive protein

Variable	Baseline	12 weeks	24 weeks	Change
CESDR total score	42	39	34	-8 pts
PAS total score	23	20	17	-6 pts
UPDRS total score	24	26	33	+9 pts
Triglycerides, mg/dL	127	70	58	-69 pts
HDL, mg/dL	44	43	48	+4 pts
Hs-CRP, mg/L	1.29	1.09	0.64	-0.65 pts
Fasting insulin, mIU/L	18.6	7.6	5.6	-13 pts
HbA1C, %	6.7	5.7	5.6	-1.1 pts
Cardiac risk ratio (Tri/HDL)	2.88	1.62	1.20	-1.68 pts
Weight, lbs	202	181.5	172.4	-29.6 pts
Waist circumference, inches	46	41.25	40	-6 pts

Depression scale: Center for Epidemiologic Studies Depression Scale-Revised (CESDR-R-20)

The participant scored a total score of 42 on the depression scale, indicating baseline symptoms of depression, including feelings of loneliness, sleep disturbances, feelings of failure, difficulty with concentration, and difficulty with motivation. According to Radloff (1977), the developer of the CESD-R-20 scale, individuals scoring above 16 were at risk of developing clinical depression [17]. A comparison of baseline, 12-week, and 24-week scores on the CESDR-R-20 scale is presented in Table 1. There was some reduction in scores over 24 weeks; however, the significance of this reduction in scores is unknown. There was also a mild reduction in symptoms of depression reported over the 24 weeks (Table 1).

Anxiety scale: the Parkinson's Anxiety Scale (PAS)

The participant scored a total score of 23 on the PAS at baseline, indicating difficulties with occasional feelings of anxiety or nervousness, tension or stress, inability to relax, and excessive worrying, with occasional heart palpitations. The maximum possible score on the PAS is 48 [10]. Higher scores on the PAS indicate greater incidences of anxiety in daily life. The participant appeared to be at moderate risk for anxiety symptoms at baseline. A comparison of baseline (score: 23), 12-week (score: 20), and 24-week (score: 17) scores on the PAS is detailed in Table 1. It is evident from the participant reports that there was some reduction in symptoms of anxiety over 24 weeks; however, the significance of the decrease in total scores was not statistically quantifiable (Table 1).

The Unified Parkinson's Disease Rating Scale (UPDRS) scores (parts I, II, and III)

The participant reported behavioral symptoms, including mild feelings of depression and vivid dreaming. She complained about unpredictable "off" periods during the day but no painful dyskinesias. She also complained at baseline of occasional anorexia, vomiting, and sleep disturbances. Gait difficulties were observed in the form of a mild tendency towards retropulsion, with a mild slowing in leg agility. Tremors in the upper extremities were observed (baseline score: moderate) but were reported to have improved at 12 weeks (mild). The most significant symptoms were reported to be painful sensations in the lower extremities, which were reported to be moderately improved at 12 weeks (mild) with no further reported improvement in scores at 24 weeks. Activities of daily living were reported to be mildly slower but independent, requiring no assistance. Question #22 on the UPDRS (rigidity) score was omitted from baseline, 12-week, and 24-week analysis as the Zoom appointment format did not allow for a "hands-on" evaluation of rigidity. The participant reported that she thought the symptoms of rigidity had subjectively improved over the 24 weeks (Table 1).

Noticeable and reported improvements in part I: mentation and behavior scores were seen after the first 12 weeks, with improvements in concentration and a reduction in symptoms of depression on the UPDRS. According to 24-week scores, these improvements were sustained, but no further reduction was noted (Table 1).

Dietary compliance was confirmed upon a review of ketone levels from baseline, and weeks 12 through week 24, with an average blood ketone reading of 0.5 mmol indicating sustained nutritional ketosis [12].

Biomarkers of health

The initial assessment of commonly tested biomarkers of health indicated that the participant qualified for the diagnosis of metabolic syndrome (low HDL, high HbA1C, high blood pressure, waist circumference >35", and BP >140/90 mmHg). The participant's results indicated that she had significant difficulty with blood glucose regulation (HbA1C of 6.7%) prior to the start of the study, as she was scoring in the range consistent with a diagnosis of diabetes [13], with average fasting glucose at 135 mmol (per baseline KetoMojo device reading). Additionally, the participant's fasting insulin was high (18.6 mIU/L), indicating difficulty with regulating blood insulin levels. Significant improvements in blood glucose control (HbA1C), and reduced fasting insulin levels were noted after 24 weeks (Table 1).

HDL cholesterol was tested at baseline and again at 12 weeks and 24 weeks. A slight rise in HDL levels was noted, which was not statistically calculable (Table 1). In addition, a decrease in CRP (marker of inflammation) readings was also noted, but the significance of this change was not statistically demonstrable. Triglycerides were significantly reduced from baseline at 24 weeks with a drop of 69 pts. Triglycerides are used in the calculation of the cardiac risk ratio (CRR) (triglycerides/HDL) [18]. Initially, the participant's CRR indicated a slightly increased risk (Trig/HDL=2.88). The 24-week CRR was 1.20, indicating a risk reduction, with <1.0 indicating average risk [19].

Waist circumference and weight were measured at baseline, 12 weeks, and 24 weeks with a significant reduction noted in waist circumference (-6") and weight (-29.6 lbs) at 24 weeks. Although the participant still had a waist circumference in the range that would fulfill one of the five criteria for metabolic syndrome (>35" for women), given the drop in her other markers including reduced HbA1C, reduced waist circumference, and reduced BP, she no longer met the criteria for a metabolic syndrome diagnosis.

Participant comments

A post-24-week study-period interview was held with the participant via Zoom. She reported she had not added much fat over the 24 weeks, but prioritized protein, added some fats, and kept carbohydrates low. She reported having more energy and attending to tasks for longer periods (she is a Ph.D. student). In addition, she reported having no cravings for sugary foods and not being hungry, which were commonly reported symptoms at baseline. Calorie limitations are a typical result of increased satiety. She now felt it was indulgent to eat bacon and sour cream, claimed her clothes fit better, and she no longer craved carbohydrates like crackers and cookies. She reported she had some issues with constipation early on in the 24-week period but had solved it with daily consumption of bone broth and increased magnesium supplementation. Towards the end of the first 12 weeks, as an incidental finding, the participant had a repeat bone density study ordered by her PCP, which had revealed improved bone density readings with a higher Z score over her pre-study baseline readings. Additionally, she expressed the view that this was the most manageable plan for losing weight that she had ever done and that she liked all the foods on the accepted list and felt satisfied with decreased hunger.

## Discussion

Nutrition studies have often received flak for their lack of credibility due to variations in recording quantities of foods and the subjective nature of the frequency of the various food regimen methods. Using biomarkers can enable a more objective assessment of food consumption and virtually eliminate bias using self-reported dietary assessments [20]. This case study utilized biomarker assessment at baseline, 12 weeks, and 24 weeks and attempted to assess dietary compliance through blood glucose and ketone-testing results. The use of a KetoMojo home blood glucose/ketone meter further assisted in validating dietary compliance [21].

Additionally, the nutritional content of various foods as documented in nutrition databases can be inconsistent due to multiple variables and may not reflect the exact nutrient content. Therefore, the use of biomarker assessment can further confirm nutritional composition, as in the case of our study participant. The combination of both a food-tracking tool, in this case, the MyFitnessPal app (Under Armour, Inc., Baltimore, MD) and biomarkers of health can provide valuable instruments for estimating nutrient intake and assessing health risks [20]. One example entails assessing fasting plasma glucose levels and associations with improved insulin sensitivity or plasma triglycerides and the link to cardiovascular disease [20].

According to a meta-analysis by Qiu et al. (2019), PD can be associated with an increase in CRP levels, potentially revealing a link between increased inflammation as measured by CRP and the development of PD [21]. Our participant's CRP was slightly higher at baseline (1.29 mg/L) than at 12 weeks (1.09 mg/L), which then further decreased at 24 weeks (0.64 mg/L). Even though her CRP levels were not significantly elevated at baseline, the 24-week results did indicate a reduction in this marker of inflammation. According to Loefler et al. (2015), in addition to motor signs and symptoms, people with PD can suffer from autonomic dysfunction and poor emotional regulation resulting in apathy, depression, and anxiety. Our participant demonstrated a modest reduction in symptoms of depression and anxiety after the 24-week dietary intervention.

The LCHF nutritional approach has been demonstrated to result in cognitive improvements in PD [22]. The diet mainly consists of meat, fish, poultry, low-carb vegetables, coconut oil, and avocados, representing nutrients such as omega-3 fatty acids and vitamins A, D, and B complex. The LCHF/KD has been used to generate blood ketones, improve neuronal cell energy and efficiency in brain neurochemistry, and produce anti-inflammatory and antioxidant properties [23]. Furthermore, clinical research studies on ketones have reported the production of blood ketones as a result of either an LCHF/KD or exogenous ketone salts or ketone esters, resulting in improved cognitive function, amelioration of symptoms in PD related to dopamine deficits, improved pain and mood, and reductions in urinary frequency [24].

Since the LCHF/KD is known to mimic fasting through the production of blood ketones, nutritional ketosis created by the diet can improve insulin sensitivity, increase supplies of brain neurotransmitters and other metabolic precursors for synaptogenesis, and reduce apoptosis [18]. According to Krikorian et al. (2019), ketone levels were positively correlated with performance on memory tests along with improvements in waist measurements, fasting glucose, and fasting insulin [23]. These results are consistent with our findings. An increase in blood ketones and reduction in other biomarkers and hypothesized changes in the participant's symptoms may account for our participant's reported improvement in behavior and mood, decreases in symptoms of both anxiety and depression, and improved cognition during the 24-week intervention.

Some of the limitations of this case report include the small sample size (n=1), non-generalizable results with a lack of statistical analysis, possible placebo effect that may have influenced participant responses, and the potential effects of weight loss that may cloud the interpretation of the effects of the dietary intervention on PD symptoms. Nutritional studies have inherent issues related to the validity of food tracking and the veracity of dietary compliance. This case report attempted to control for these variables with daily food tracking by using a widely used daily food tracking app (MyFitnessPal) and home blood glucose/ketone testing.

## Conclusions

The implications of the use of dietary interventions in PD can be significant. In the face of rising healthcare costs, improving outcomes for patients with PD through a nutritional approach when medications may not offer sustained improvements or control of symptoms can provide a more natural method for symptoms management and enhance the quality of life. Empowering individuals with PD through a more personalized nutritional approach to symptom management can be achieved by offering adjuvant treatment approaches together with medication therapy to reduce symptoms and improve function. The results of this case study (n=1) demonstrate the need for more randomized clinical trials to further test the effectiveness of the LCHF/KD in improving cognitive function and controlling or reducing symptoms of depression, anxiety, and both motor and nonmotor symptoms in PD.
